# Do emergency medical dispatchers choose the same response to serious injury in men and women – a qualitative study

**DOI:** 10.1186/s12873-024-00985-0

**Published:** 2024-04-13

**Authors:** Marlene Mellum, Raika Saei, Guttorm Brattebø, Torben Wisborg

**Affiliations:** 1 Faculty of Health Sciences, Interprofessional Rural Research Team-Finnmark, University of Tromsø - the Arctic University of Norway, Hammerfest, Norway; 2 Norwegian National Advisory Unit On Emergency Medical Communication, Department of Anaesthesia & Intensive Care, Haukeland University Hospital, Bergen, Norway; 3 Department of Clinical Medicine, University of Bergen, Bergen, Norway; 4 Norwegian National Advisory Unit On Trauma, Division of Emergencies and Critical Care, Oslo University Hospital, Oslo, Norway; 5 Department of Anaesthesiology and Intensive Care, Hammerfest Hospital, Finnmark Health Trust, Hammerfest, Norway

**Keywords:** Sex, Gender differences, Emergency medical dispatcher, Emergency medical services, Trauma, Trauma team activation, First response

## Abstract

**Background:**

Recent research has indicated that sex is an important determinant of emergency medical response in patients with possible serious injuries. Men were found to receive more advanced prehospital treatment and more helicopter transportation and trauma centre destinations and were more often received by an activated trauma team, even when adjusted for injury mechanism.

Emergency medical dispatchers choose initial resources when serious injury is suspected after a call to the emergency medical communication centre. This study aimed to assess how dispatchers evaluate primary responses in trauma victims, with a special focus on the sex of the victim.

**Methods:**

Emergency medical dispatchers were interviewed using focus groups and a semistructured interview guide developed specifically for this study. Two vignettes describing typical and realistic injury scenarios were discussed. Verbatim transcripts of the conversations were analysed via systematic text condensation. The findings were reported in accordance with the Consolidated Criteria for Reporting Qualitative Studies (COREQ) checklist.

**Results:**

The analysis resulted in the main category “Tailoring the right response to the patient”, supported by three categories “Get an overview of location and scene safety”, “Patient condition” and “Injury mechanism and special concerns”. The informants consistently maintained that sex was not a relevant variable when deciding emergency medical response during dispatch and claimed that they rarely knew the sex of the patient before a response was implemented. Some of the participants also raised the question of whether the Norwegian trauma criteria reliably detect serious injury in women.

**Conclusions:**

The results indicate that the emergency medical response is largely based on the national trauma criteria and that sex is of little or no importance during dispatch. The observed sex differences in the emergency medical response seems to be caused by other factors during the emergency medical response phase.

**Supplementary Information:**

The online version contains supplementary material available at 10.1186/s12873-024-00985-0.

## Background

In most countries, it is expected that all inhabitants have the same access to health care. Nonetheless, some studies have shown that individual factors, such as sex, may influence triage, prehospital treatment, transport destination and trauma team activation even when serious injury is suspected [1, 2]. A recent study from Stockholm showed that sex was an important individual determinant of emergency medical response in patients with road traffic injuries [1]. Similar findings were made in a study from Toronto [2].

### Sex as a variable

It has been speculated that the trauma mechanism could be a reason for men obtaining a higher prehospital priority than women [1, 2]. The study from Stockholm showed that the most common mechanism of injury for both sexes was road traffic-related injuries, while the second most common mechanism for women was falls with low energy and falls with high energy for men [1]. One could speculate that the energy involved in the trauma mechanism is highly prioritised during triage, which might result in disparities in dispatch priority and resources allocated to male versus female patients [1, 2]. Nevertheless, the study from Stockholm showed that men had a 2.75-fold greater odds ratio (OR) of receiving high prehospital priority during transportation from scene than women did, even after adjusting for the mechanism of trauma [1].

The study from Toronto showed that women, in comparison with men, had an OR of 0.88 for being transported to a trauma centre if assessed by emergency medical system (EMS) providers on scene and an even lower OR (0.85) if assessed by a physician at the primary hospital. In both cases, the patients were seriously injured, and the authors adjusted for confounding factors. This suggests that women with injuries of the same severity are less likely than men to be transported to a trauma centre from the site of injury or as a secondary transfer [2].

In the Norwegian trauma system dispatchers at the Norwegian Emergency Medical Coordination Centres (EMCCs) decide whether to dispatch ordinary road ambulances or advanced road or airborne resources based on their interpretation of the situation after assessing information from the caller. As helicopters and advanced road units are based at trauma centres and major hospitals it is conceivable that hospital destination and trauma team activation will be partly determined by the decisions made during dispatch, as well as advanced treatment on the site of injury will only be given if advanced prehospital personnel is dispatched.

### Triage, trauma, and trauma centre

**Fig. 1:**
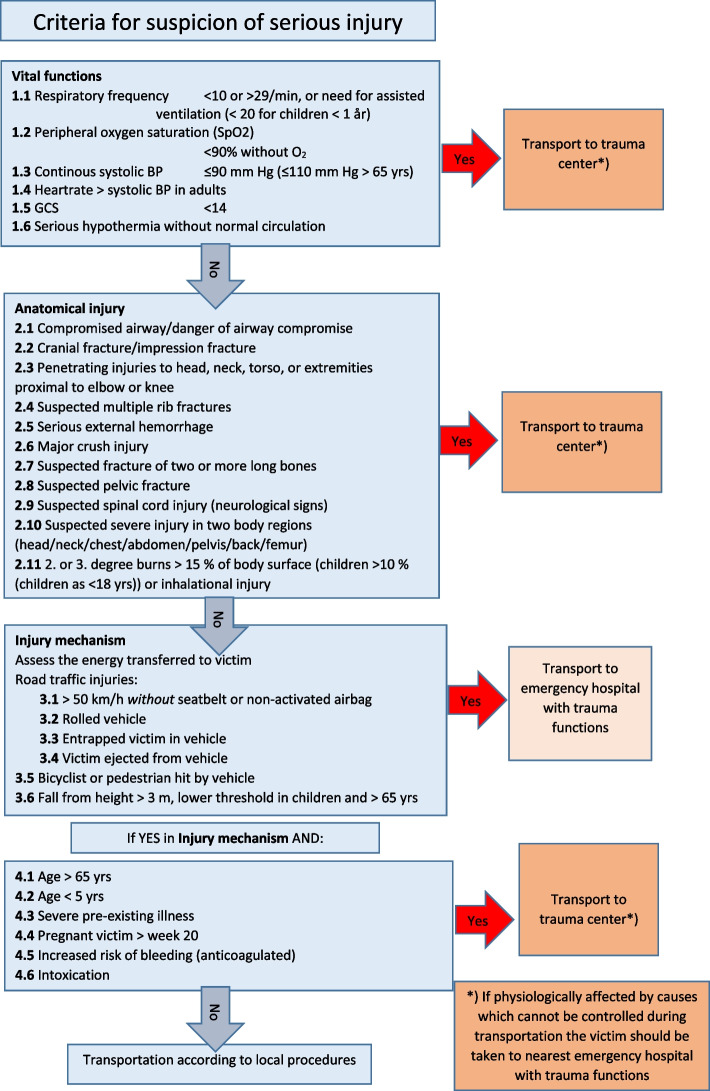
The criteria for identification of serious injury were obtained from the Norwegian National Trauma Plan [3]

The Norwegian National Advisory Unit on Trauma is a governmental organisation establishing criteria for the identification of serious injury and criteria for trauma team activation. A match in the trauma criteria should activate a trauma team (Fig. 1), and the patient is received by a trauma team in a designated trauma hospital [3].

Norway has 34 hospitals with trauma functions and four trauma centres [4].

Studies have shown that the mortality and morbidity are lower in patients treated directly at a trauma centre and by a trauma team than in patients treated at a hospital without trauma [5]. This also applies to Norway [6].

### Norwegian emergency dispatch centres

Sixteen Norwegian Emergency Medical Coordination Centres (EMCCs) respond to the national emergency phone number. They initiate acute medical responses, give advice and guidance, and prioritise and coordinate. The EMCC commands the fleet of ground ambulances, air ambulances (helicopter and fixed-wing), physician-manned rapid response cars, special ambulances, emergency first responders, and boat ambulances.

Unlike in other countries, Norwegian EMCCs are manned by nurses and ambulance personnel with special competence in handling medical emergencies. They will synchronously assign the proper units and professionals to the scene and coordinate other necessary efforts, including instructions to the caller in first aid measures. Medical dispatchers primarily use the national trauma criteria as a decision tool in trauma cases but still need to make their own professional evaluations in every situation [3]. For non-trauma calls the dispatchers use Norwegian Index for Emergency Medical Dispatch [7]. All EMCCs have an emergency medicine physician available for consultation by medical dispatchers. The initial evaluation by the dispatcher in the EMCC will determine several of the following actions: If an ambulance helicopter (with anaesthesiologist) is dispatched, it will usually be based at a trauma centre, and thus the chance of returning to this centre will increase. Advanced treatment at the scene will be given only if an air ambulance helicopter or an anaesthesiologist-manned rapid response car is dispatched. This might happen as a rendezvous, but due to long distances the primary responding vehicle might start transportation instead of waiting for the anaesthesiologist. Due to the essential role of emergency medical dispatchers as the first contact point with the health care system when serious injury is suspected, we aimed to examine which factors dispatchers see as important when handling emergency calls concerning injuries, specifically whether sex is one of them.

## Methods

This study aimed to determine the response of Norwegian emergency medical dispatchers to suspected severe injury. The decisions made by the dispatcher are based on interpretation of information from the caller, and although supported by decision support algorithms, the process involves a high amount of individual assessment and mental evaluation. To understand these processes and determinants of response, a qualitative approach was deemed the most appropriate for obtaining in-depth insights [8].

The primary interest was whether the sex of the victim influenced the choice of response.

### Study design

We employed semistructured focus group interviews with dispatchers at their working place and based the conversations on an interview guide with illustrative vignettes. Focus groups were chosen because dispatchers usually work individually, and we wanted collective experiences among informants [9].

The study adhered to the Consolidated Criteria for Reporting Qualitative Studies (COREQ): 32-item checklist [10] (Supplemental file 1).

Vignettes were realistic cases based on frequent mechanisms of injury. They were constructed to not directly fit the national trauma criteria, ensuring that the participants would discuss the cases more freely (Supplemental file 2).

The interview guide was structured according to Kvale and Brinkmann's model for designing interview questions in qualitative interviews, and was developed specifically for this study (Supplemental file 2) [11]. The interviews started with open-ended questions, and direct questions were postponed until the end of the interview so that "…the interviewees get to give their own, spontaneous descriptions first" [11].

Focus group interviews were performed in person by one of the authors (MM or RS, both female medical students), while the other author took notes. The authors had no contact with the participants prior to the interviews. No nonparticipants were present during the focus group interviews. All interviews were audio recorded.

After inviting participants to discuss the vignettes, we introduced different variables shown in the literature to be the most important factors for different dispatch and prehospital treatment decisions for further discussion. These factors were age, geography, and sex [1, 2, 5, 12, 13]. Sex was deliberately postponed and introduced only after the participants agreed on the emergency response and dispatch.

### Recruitment of participants

We recruited participants by contacting the leaders of four large EMCCs in Norway. The EMCCs were chosen based on their geographical location, number of employees, number of calls per year, and capacity. We continued recruitment until no new information emerged after the last interviews, i.e., data saturation was reached [14]. This is a well-known term in qualitative research that describes a point where the collection of more data will not bring the research any new information.

Participants in each focus group were chosen by convenience sampling. We aimed to recruit medical dispatchers of different backgrounds to obtain different points of view in the discussions. In total, we interviewed 18 individuals (10 women and eight men). Twelve were registered nurses, and the rest were ambulance personnel. The participants had one to 20 years of EMCC experience. To obtain the participants’ points of view, we did not limit the duration of the interviews. On average, they lasted approximately one hour.

### Analysis of the material

The material was analysed in two rounds; transcription followed by systematic text condensation. Transcription and analysis were performed after each focus group interview to allow for adjustments in the interview guide before the next interview.

Systematic text condensation, which is a four-step analysis method based on a cross-sectional analysis of the material, was used for analysis [14]. The analysis was divided into the formation of an overall impression, selection of meaningful units, condensation, and synthesis. The two first authors performed the first analysis, whereafter all authors discussed the findings.

## Results

**Fig. 2:**
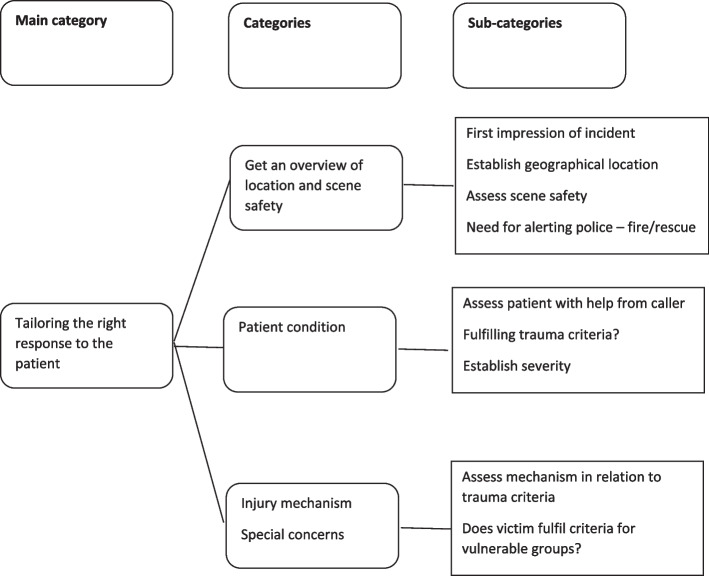
Emergency medical dispatschers’ initial priorities when deciding response in trauma alert calls. Subcategories and categories that make up the main category

The analysis resulted in the main category “Tailoring the right response to the patient”, supported by three categories: “Obtain an overview of location and scene safety”, “Patient condition” and “Injury mechanism and special concerns”. The main category, categories and subcategories are presented in Fig. 2. They are described and illustrated with relevant quotations below.

### Obtain an overview of location and scene safety

The informants stated that they primarily tried to get an overview concerning geographical location and safety at the scene. “*Most often, when we are told about trauma, it is not the patient we are talking to but usually the public. Then, we know that we are talking about obvious trauma and serious incidents”* [EMCC 2, informant 3]. *"In the case of traffic accidents, it is important to ensure the scene of the accident and the safety of the caller.”* [EMCC 1, informant 2]. This allows an assessment of the need for sending police and fire/rescue services as well as ambulances.

The informants underlined that the response was determined by an overall assessment of the situation. *“… it’s not one single piece of information or finding that determines the response. It’s more the total picture”* [EMCC 2, informant 2].

### Patient condition

After these first assessments, the informants described probing for patient information. *«Primarily, the national trauma criteria determine our response… If we suspect a serious injury, we may deviate from the written template and focus on the patients’ condition» [EMCC 4, informant 1].*

There seemed to be a mutual understanding that road traffic collisions often would receive the highest priority initially, of the injury mechanism, suspected injuries, or speed limitations at the scene. «*We probably have a culture for increasing the response initially and then scaling down afterwards when more information occurs». [EMCC 4, informant 2].* Despite large geographical variations between EMCCs, the use of air ambulances seemed to be assessed similarly. *«We’ll send one or more (road) ambulances, prehospital physician and air ambulance while collecting information, and afterwards, the air ambulance assesses the need by themselves… The need for activating the air ambulance is often based on the EMCC operator’s experience» [EMCC 1, informant 5].*

Several informants noted that better adherence to the national trauma triage criteria has made them more independent of consulting with EMCC physician. *«The later years we have been better at using the trauma criteria… Now we follow the criteria strictly and activate the trauma team when they are fulfilled. The criteria are needed to secure equal treatment for all»*. [EMCC 4, informant 2].

The informants were concerned about the patient’s status and vital functions. *«Mainly the ABC and consciousness, which should be clarified initially. How is the GCS status, and has it changed? What does the caller report about pain, and does the patient belong to the vulnerable groups defined in the national trauma plan?»* [EMCC 1, informant 4].

### Injury mechanism and special concerns

Several informants underlined that while the individual patient was assessed according to the national trauma guidelines, they also focused on mechanisms and special concerns.

The time of day was one concern for the informants. “*The time of day is important. It is … during the night hours we see much driving under the influence… this should be taken into consideration as it may cover physical findings… I have a feeling that the most serious traffic-related injuries are during the night”* [EMCC2, informant 7].

A lack of trustworthy information was considered an indication of increased attention and a lowered threshold for dispatch.*»I am thinking of the fact that the less we know, the more we need to assure that the patient gets the help needed*» [EMCC 3, informant 1]. Another informant added that the patients would receive help anyway,*»…but without undue delays. I think we send more resources the less we know?*» [EMCC 3, informant 2].

The mechanism of injury was highlighted by several informants. This was especially important in road traffic scenarios. *«The injury mechanism is important. There is a large difference between front-to-front collisions and driving off the road. It’s important to ascertain whether any victims are entrapped, as this is often linked to severe injury»* [EMCC1, informant 1].

### Does sex influence emergency response?

At the conclusion of the focus group interviews, the informants were directly asked about the possible influence of the victim’s sex. All dispatchers consistently denied that sex was important for handling dispatch in these patients.

Most informants said that they rarely know age or sex until they are well involved in the process:*»Sex is irrelevant to how we choose our response, and we very rarely know the sex of the patients involved before they have arrived at the hospital*»*.* [EMCC 2, informant 5].

Some informants noted that the only basis for handling women and men differently was if it was conceivable that the woman was pregnant and thus, based on the trauma criteria, was defined as being more vulnerable. At the same time, they explained that they most often talk to bystanders in trauma cases and not with the patient itself, so sex is rarely revealed at the first response.*»Most often, when we are told about trauma, it is not the patient we are talking to but usually the public. Then, we know that we are talking about obvious trauma and serious incidents. A woman, on the other hand, can be pregnant; then, you have two patients. Then, men tend to tolerate a lot more due to muscle mass and physiological conditions, but this will not affect the response based on sex in itself if everything else is equal*»*.* [EMCC 2, informant 3].

It was also discussed whether the trauma criteria used in Norway today are sufficient to include both women and men.*»Sex probably has no bearing on our conscious response, but what I am more concerned about is whether the index actually captures serious injuries in women. We know that some medical conditions are not caught because all the parameters we use have been researched on men, so is it conceivable that we simply do not catch the women with the trauma criteria we have?*» [EMCC 3, informant 1].

## Discussion

We found that the informants consistently did not emphasise the victims’ sex during the assessment or decision of response.

This theme is difficult to survey objectively due to societal and culturally "correct" perceptions, although all measures were taken to avoid such confounders. We employed a method using realistic vignettes and did not provide information about the study aim concerning victims’ sex until the end of each focus group interview.

It appears, in both vignette discussions and from the focus groups at large from all EMCCs, that sex is not initially important for the choice of response. Several of the informants noted that they often do not know the sex of the patient before a response is initiated. Nevertheless, it was noted in both situations described in the vignettes that women might be pregnant and thus defined as a "vulnerable group". One of the interviews also highlighted by an informant that men are perhaps "more robust physiologically and anatomically" than women are. Based on these statements, it is conceivable that women would receive a greater EMCC-initiated prehospital response than men. This finding contrasts with the findings in the literature [1, 2]. However, this difference does not seem to stem from prejudice or active will of mind during the dispatch phase.

### Possible causes of different EMCC responses in relation to sex

When several studies have shown different responses between women and men [1, 2]; even when controlling for injury mechanisms, exploring the handling of emergency calls is natural. The EMCC is the first contact point between the public and the emergency and health care system, and critical decisions are made in the dispatch phase. Our findings contradicted the conscious differentiation between male and female victims in the dispatch phase.

### The national trauma criteria

Some of the participants raised questions regarding whether the national trauma criteria are designed to capture serious injury in women. According to the National Trauma Plan and the Norwegian National Trauma Registry, the knowledge base for the criteria is comprehensive and solid, while "systematic review of patients who are over- and undertriaged for possible changes to the criteria" is recommended [15]. No Norwegian studies have assessed whether men are overrepresented in the overtriage group. The studies on which our project is based all conclude that there is a sex difference in the handling of seriously injured patients, but it would also be interesting to investigate whether the difference is due to an overtriage of men rather than an undertriage of women.

Some of the participants noted that physiological impact is used to assess the severity of injuries according to today's trauma criteria. These data include respiratory rate, heart rate, SpO_2_, systolic blood pressure, Glasgow Coma Scale (GCS) score, and temperature. The challenge with these guiding triage criteria is that the studies from which they are derived are based on previous cohorts of trauma victims, and need revisions when trauma victims and mechanisms change [16]. Among other factors, we know less about women's compensatory mechanisms in severe injury. This topic deserves additional research before the trauma criteria can be adjusted.

### Limitations

This study was based on focus group interviews. Four EMCCs participated, and despite purposeful sampling of EMCCs there is a risk that other informants might have had other views. The similarity of information from all EMCCs indicate that the findings are at least representative for the centrals interviewed. As the centrals were diverse geographically and in size, we believe that the findings are representative despite a limited number of informants.

The authors were aware of the risk of societal and culturally "correct" perceptions. Due to this, all measures were taken to introduce sex as a study aim only after the informants reached a common decision concerning dispatch of resources. Still, there is a possibility that some informants may have chosen answers meant to please the investigators or socially acceptable norms.

## Conclusions

This study investigated whether the sex of a suspected severely injured patient is important during the dispatch phase when emergency medical dispatchers must choose a suitable response. The results indicate that the response is largely based on the national trauma criteria.

We can safely say that our informants do not seem to consciously handle patients differently based on sex. The informants reported that one possible cause for sex-related differences could be differences in physiological responses after severe injury between the two sexes. This topic requires further research and may have an impact on the use of the trauma criteria in the future.

### Supplementary Information

**Supplementary Material 1.**

**Supplementary Material 2.**

## Data Availability

The interviews are not available for distribution due to restrictions in the interest of confidentiality.
