# Supplementary material for: Do emergency medical dispatchers choose the same response to serious injury in men and women – a qualitative study
Source: BMC Emerg Med. 2024 Apr 13;24:60. doi: 10.1186/s12873-024-00985-0 (PMC11015548; doi:10.1186/s12873-024-00985-0)
Supplement: Supplementary file 2 — Supplementary Material 2. [file 12873_2024_985_MOESM2_ESM.docx]

Supplementary file 2

Interview guide

It is intended that informants are discussing between themselves based on this semi-structured interview guide.

Demographics:

Are the informants nurses or paramedics (or both)?

How long have the informants worked as EMCC operators?

Introduction:

The informants were introduced to the first vignette and asked to discuss how they would choose to respond and what additional information they would ask for to make this decision.

Supplementary questions:

What would be your first thoughts on this situation?

How do you handle this emergency call onwards?

Which variables or information helps you decide upon a response?

What information from caller do you consider most important?

Vignettes:

First vignette: The caller reports a traffic incident involving one car. Damage to the vehicle; two people were involved. This happened in an area with a posted speed limit of 60 km/h.

Second vignette: The caller reports a person who falls from a ladder. This happened outdoors. The person landed on a grass lawn.
